# Retinal Degeneration Associated With RPGRIP1: A Review of Natural History, Mutation Spectrum, and Genotype–Phenotype Correlation in 228 Patients

**DOI:** 10.3389/fcell.2021.746781

**Published:** 2021-10-14

**Authors:** Avigail Beryozkin, Hamzah Aweidah, Roque Daniel Carrero Valenzuela, Myriam Berman, Oscar Iguzquiza, Frans P. M. Cremers, Muhammad Imran Khan, Anand Swaroop, Radgonde Amer, Samer Khateb, Tamar Ben-Yosef, Dror Sharon, Eyal Banin

**Affiliations:** ^1^Department of Ophthalmology, Hadassah Medical Center, Faculty of Medicine, The Hebrew University of Jerusalem, Jerusalem, Israel; ^2^Genetics, Biomedical Department, Faculty of Medicine, Universidad Nacional de Tucumán, Tucumán, Argentina; ^3^Ophthalmology, Clinical Department, Faculty of Medicine, Universidad Nacional de Tucumán, Tucumán, Argentina; ^4^Neurology, Clinical Department, Faculty of Medicine, Universidad Nacional de Tucumán, Tucumán, Argentina; ^5^Department of Human Genetics, Radboud University Medical Center, Nijmegen, Netherlands; ^6^Neurobiology-Neurodegeneration and Repair Laboratory, National Eye Institute, National Institutes of Health, Bethesda, MD, United States; ^7^The Ruth and Bruce Rappaport Faculty of Medicine, Technion-Israel Institute of Technology, Haifa, Israel

**Keywords:** retinal degeneration, RPGRIP1, natural history, mutation spectrum, genotype-phenotype correlation

## Abstract

**Purpose:** *RPGRIP1* encodes a ciliary protein expressed in the photoreceptor connecting cilium. Mutations in this gene cause ∼5% of Leber congenital amaurosis (LCA) worldwide, but are also associated with cone–rod dystrophy (CRD) and retinitis pigmentosa (RP) phenotypes. Our purpose was to clinically characterize *RPGRIP1* patients from our cohort, collect clinical data of additional *RPGRIP1* patients reported previously in the literature, identify common clinical features, and seek genotype–phenotype correlations.

**Methods:** Clinical data were collected from 16 patients of our cohort and 212 previously reported *RPGRIP1* patients and included (when available) family history, best corrected visual acuity (BCVA), refraction, comprehensive ocular examination, optical coherence tomography (OCT) imaging, visual fields (VF), and full-field electroretinography (ffERG).

**Results:** Out of 228 patients, the majority (197, 86%) were diagnosed with LCA, 18 (7%) with RP, and 13 (5%) with CRD. Age of onset was during early childhood (*n* = 133, average of 1.7 years). All patients but 6 had moderate hyperopia (*n* = 59, mean of 4.8D), and average BCVA was 0.06 Snellen (*n* = 124; only 10 patients had visual acuity [VA] > 0.10 Snellen). On funduscopy, narrowing of blood vessels was noted early in life. Most patients had mild bone spicule-like pigmentation starting in the midperiphery and later encroaching upon the posterior pole. OCT showed thinning of the outer nuclear layer (ONL), while cystoid changes and edema were relatively rare. VF were usually very constricted from early on. ffERG responses were non-detectable in the vast majority of cases. Most of the mutations are predicted to be null (363 alleles), and 93 alleles harbored missense mutations. Missense mutations were identified only in two regions: the RPGR-interacting domain and the C2 domains. Biallelic null mutations are mostly associated with a severe form of the disease, whereas biallelic missense mutations usually cause a milder disease (mostly CRD).

**Conclusion:** Our results indicate that *RPGRIP1* biallelic mutations usually cause severe retinal degeneration at an early age with a cone–rod pattern. However, most of the patients exhibit preservation of some (usually low) BCVA for a long period and can potentially benefit from gene therapy. Missense changes appear only in the conserved domains and are associated with a milder phenotype.

## Introduction

Leber congenital amaurosis (LCA) is considered as the most severe form of non-syndromic inherited retinal degeneration. It is usually diagnosed during the first months of life and leads to severe visual impairment or total blindness already at an early age. It is characterized by oculo-digital signs, congenital nystagmus, hypermetropia, and non-recordable electroretinography (ERG) responses (Cremers et al., 2002; den Hollander et al., 2008; Kumaran et al., 2017). LCA is a genetically heterogeneous disease, and to date, 14 causative genes were identified for LCA (RetNet -- Retinal information Network^1^); one of these is *RPGRIP1* (retinitis pigmentosa [RP] GTPase regulator interacting protein), which is responsible for approximately 5–6% of all LCA cases (Dryja et al., 2001; Gerber et al., 2001).

The *RPGRIP1* gene maps to the 14q11.2 chromosomal region and encodes several different isoforms with distinct cellular, sub-cellular, and biochemical properties (Koenekoop, 2005). The longest isoform consists of 24 exons and is predicted to encode a protein product of 1,287 amino acids (Gerber et al., 2001; Koenekoop, 2005). The RPGRIP1 protein contains several domains: a coiled-coil domain, homologous to proteins involved in vesicular trafficking located at the N-terminus; two C2 domains, which are usually involved in targeting proteins to the cell membrane; a bipartite nuclear localization domain; a C-terminal RPGR-interacting domain; and two parts of seryl tRNA synthetase at the C- and N-termini (Roepman et al., 2000; Koenekoop, 2005). In addition, the RPGRIP1 protein includes many phosphorylation, glycosylation, amidation, and myristoylation sites, as well as zinc fingers and leucine zipper structures.

RPGRIP1 was first identified in the outer segments of photoreceptors (Roepman et al., 2000; Mavlyutov et al., 2002) and later was reported specifically in centrioles, basal bodies (Shu et al., 2005), and photoreceptor connecting cilium (Hong et al., 2001). RPGRIP1 expression was also reported in amacrine cells (Castagnet et al., 2003). This protein is a structural protein of the ciliary axoneme in the connecting cilium of all types of photoreceptors (Boylan and Wright, 2000; Roepman et al., 2000). RPGRIP1 has been reported to be involved in anchoring RPGR within the cilium (Roepman et al., 2000), vesicular trafficking (Roepman et al., 2000), and directional transport of nascent proteins from the inner segment destined for the outer segment (Hong et al., 2001) and participates in disc morphogenesis (Zhao et al., 2003).

A *Rpgrip1-*knockout mouse line was generated and characterized phenotypically (Zhao et al., 2003). This mouse model shows abnormal outer segments from a very early age and complete photoreceptor loss at the age of 5 months. In addition, these authors showed that RPGR, a protein that interacts with RPGRIP1, depends on RPGRIP1 for subcellular localization and normal function (Zhao et al., 2003). Successful gene therapy was performed in the mouse model and also in canine models of RPGRIP1 and was able to restore the localization of RPGR, improve photoreceptor survival, and preserve cone and rod function (Pawlyk et al., 2010; Lhériteau et al., 2014). Thus, there may be treatment potential for LCA associated with RPGRIP1.

In the current study, we attempted to characterize clinical phenotypes in a large cohort of patients harboring *RPGRIP1* mutations, integrating clinical data that were reported previously in the literature and clinical data from an Israeli cohort of patients. Our goal is to provide information on the clinical spectrum and course of disease associated with the *RPGRIP1* gene, including identification of characteristic features. The findings can assist in the evaluation and diagnosis of patients globally and provide data on prognosis and may be relevant for therapies such as gene augmentation, which has already been shown to be successful in mouse (Pawlyk et al., 2010) and dog models (Lhériteau et al., 2014).

## Materials and Methods

All methods were carried out in accordance with relevant guidelines and regulations.

### Subjects

We recruited for the study Israeli and Palestinian individuals with various inherited retinal dystrophies (IRDs), including LCA, RP, and cone–rod dystrophy (CRD). Before drawing a blood sample for molecular analysis, all participants in the study or their legal guardians signed an informed consent that adhered to the tenets of the Declaration of Helsinki. Ethical approval for this study was obtained from the Hadassah-Hebrew University Medical Center and the Rambam Health Care Center IRB committees.

### Genetic Analysis

Genetic analysis was performed by whole exome sequencing (WES) (Beryozkin et al., 2015) or molecular inverted probes (MIPs) (Neveling et al., 2017; Weisschuh et al., 2018) analysis, verified by Sanger sequencing of PCR products. Primers for all suspected variants were designed using the Primer3 online program^2^ for mutation screening of the *RPGRIP1* exons and exon–intron boundaries (NCBI Reference Sequence NM_020366.4) by PCR amplification (Supplementary Table 1). PCR was performed in a 30-μl reaction with 35 amplification cycles. The possible pathogenicity of missense changes was evaluated using PolyPhen-2^3^, MutationTaster^4^, and SIFT^5^.

### Clinical Evaluation

Clinical information was collected retrospectively at Hadassah-Hebrew University Medical Center, Rambam Health Care Center, and related research manuscripts that were published previously (Dryja et al., 2001; Gerber et al., 2001; Hameed et al., 2003; Hanein et al., 2004; Galvin et al., 2005; Zernant et al., 2005; Jacobson et al., 2007; Vallespin et al., 2007; Seong et al., 2008, 2015; McKibbin et al., 2010; Walia et al., 2010; Li et al., 2011; Abu-Safieh et al., 2013; Chen et al., 2013; Fakhratova, 2013; Huang et al., 2013, 2016, 2017; Khan et al., 2013, 2014; Verma et al., 2013; Coppieters et al., 2014; Suzuki et al., 2014; Watson et al., 2014; Boulanger-Scemama et al., 2015; Maria et al., 2015; Saqib et al., 2015; Wang et al., 2015, 2016; Abouzeid et al., 2016; Tiwari et al., 2016; Han et al., 2017; Jinda et al., 2017; Riera et al., 2017; Birtel et al., 2018; Hosono et al., 2018; Imani et al., 2018; Sanchez-Navarro et al., 2018; Avela et al., 2019; Jamshidi et al., 2019; Jespersgaard et al., 2019; Miyamichi et al., 2019; Sallum et al., 2020; Sato et al., 2020; Skorczyk-Werner et al., 2020). Clinical data included, when available, anamnestic information on disease onset, progression and different symptoms, best corrected visual acuity (BCVA), refractive error, clinical ocular exam by slit lamp biomicroscopy, full-field electroretinography (ffERG), Goldmann visual fields (VF, using the I4e, III4e, and/or V4e targets according to stage of disease), optical coherence tomography (OCT, using the Heidelberg Spectralis system), color, infrared, and fundus autofluorescence (FAF) imaging (using a Zeiss and/or Optos fundus camera and the Heidelberg Spectralis system) (Beryozkin et al., 2020).

Best corrected visual acuity was measured at each visit of the patient, and the average of both eyes (BE) was taken. In case the patient underwent cataract surgery and the BCVA improved in the operated eye, measurements prior to surgery that were lower in this eye were corrected to the measurement post-surgery with the thought that this better represents retinal function at that time. In order to provide numerical values for low BCVAs, the following conversions were made: no light perception (NLP) = 0, light perception (LP) = 0.0001, hand movement (HM) = 0.001, and finger count (FC) = 0.01. When necessary, LogMAR of BCVA was converted to the Snellen equivalent using an online converter program^6^ (Beryozkin et al., 2020).

Visual fields in previously published articles were measured using different techniques with different default definitions. We were not able to summarize and compare between those measurements. Because most of the available VF data from patients were collected using the Goldmann V4e target, this was the default target we used.

Full-field ERG responses were recorded according to the ISCEV standard using corneal electrodes and a computerized system (UTAS 3000; LKC, MD, United States) as previously described (Beit-Ya’acov et al., 2007). The average cone flicker amplitude between the two eyes as measured on the earliest ERG test performed in each of the patients was included in our analysis (age at first ERG testing may be indicative of disease severity).

### Bioinformatics’ Analysis

Protein structure, protein domains, and functional sites were identified and sketched using various online platforms: ProSite https://prosite.expasy.org/, Pfam http://pfam.xfam.org/, HaMap https://hamap.expasy.org/, UniProt https://www.uniprot.org/help/motif, and Motif Scan https://myhits.sib.swiss/cgi-bin/motif_scan.

Amino acid sequences of RPGRIP1 orthologs were extracted from the HomoloGene database at NCBI^7^. The following sequences were used for the analysis: human (NP_065099.3), chimpanzee (PNI22424.1), macaca (XP_028707222.1), dog (NP_001300702.1), cow (XP_024853050.1), rat (XP_017455358.1), mouse (NP_076368.1), frog (XP_012826764.2), and zebrafish (XP_021325946.1). Amino acid sequences were aligned with the ClustalW2 multiple alignment tool at EBI^8^. The sliding window analysis was performed as previously described (Bandah-Rozenfeld et al., 2010; Sharon et al., 2018), with a 50 amino acid interval, between the human RPGRIP1 sequence and each representative ortholog.

### Data Availability

The datasets generated and/or analyzed in the current study are available from the corresponding author upon reasonable request.

## Results

### Identification of *RPGRIP1* Mutations

Our cohort includes over 2,000 index cases with IRDs. Genetic analyses revealed biallelic *RPGRIP1* mutations in 7 index cases (a total of 16 patients), and all available clinical data from those patients were collected retrospectively. To the best of our knowledge, all the reported cases from our cohort harbored only biallelic *RPGRIP1* mutations. No mutations were identified in other IRD genes. In addition, we collected clinical data from 212 patients with homozygous or compound heterozygous mutations previously published in the literature. The data, when available, included general clinical diagnosis of the phenotype, age of first signs or symptoms, refraction, BCVA, ffERG, VF, presence of nystagmus or roving eye movements, photophobia, nyctalopia, oculodigital reflex, and presence of intellectual disability or developmental delay (Supplementary Table 2).

### MOL0358

**FIGURE 1:**
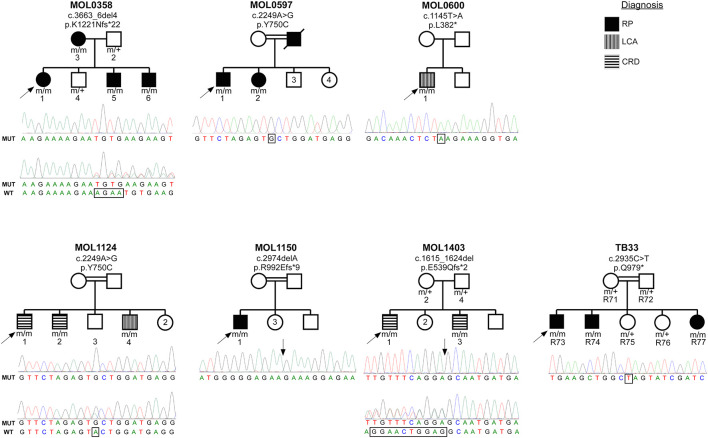
Pedigrees of the seven families with RPGRIP1 mutations in the Israeli cohort. Family number and the causative mutation (at the cDNA and protein levels) are noted above each pedigree. The number of each subject is listed below each symbol. Shapes marked in black denote an RP phenotype, horizontal stripes indicate a CRD phenotype, and the two individuals with a LCA phenotype are denoted by vertical stripes. White shapes indicate healthy family members. Index cases are marked with black arrows. Underneath each pedigree, a chromatogram of the mutated allele (and when available of a heterozygous allele) is presented. The exact locations of the mutations are marked with rectangles and arrows.

The index case (MOL0358-1) belongs to a Christian family originating from Argentina. She manifested nystagmus shortly after birth, and ffERG testing that was performed in Argentina at the age of 4 months was reported to be sub-normal. She was diagnosed with early onset RP, in similarity to her mother and two of her brothers (pedigree is detailed in Figure 1). All four patients (who belong to two generations) were found to be homozygous for c.3663_6del4 (Sharon et al., 2020) (p.K1221Nfs^∗^22), in the *RPGRIP1* gene (Figure 1).

**FIGURE 2:**
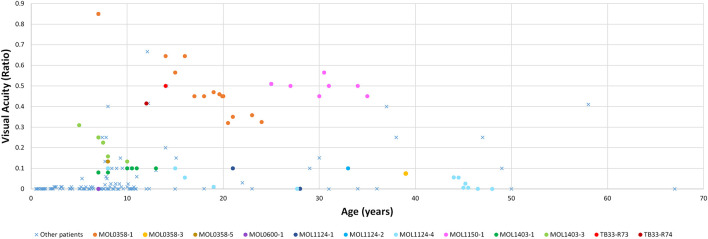
Best corrected visual acuity measurements (presented as Snellen ratio) versus age. Measurements of *RPGRIP1* patients previously reported in the literature are denoted by a blue X. The earliest available measurement for each such patient was taken. BCVA measurements of patients from our cohort are presented as circles, with each color representing a different patient. In this group, all available VA measurements along time are shown.

**FIGURE 3:**
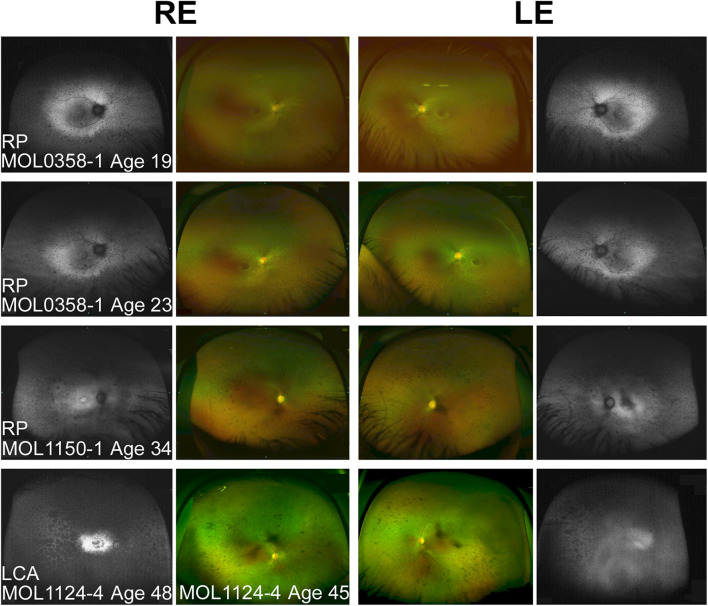
Spectrum of fundus findings among *RPGRIP1* patients from our cohort at different ages. Wide field OPTOS pseudocolor and fundus autofluorescence (FAF) images show that bone spicule-like pigmentary changes appear at teen years. In older patients (ages 30+), nummular pigmentation was also observed. Diagnosis, patient numbers, and the age at the time of imaging are specified on each of the panels. FAF images of MOL1124-4 from the age of 45 were not available because of technical problems, and FAF images at the age of 48 are shown instead. The FAF image of the left eye of this patient is partially obscured because of cataract.

Patient MOL0358-1 first presented to our clinic at Hadassah-Hebrew University Medical Center at the age of 7 years, and repeated ffERG testing at that time revealed non-detectable responses under both photopic and scotopic conditions. Her visual acuity (VA) was relatively well preserved, and she maintained a BCVA of 0.63 up to the age of 16 (Figure 2). Later on, her BCVA gradually deteriorated, reaching 0.4 in the right eye (RE) and 0.25 in the left eye (LE) on the last exam performed at the age of 24. Her refraction was +2.50/−2.75 × 5° in the RE and +1.75/−2.50 × 160° in the LE at the age of 19 years (Supplementary Table 2). Intraocular pressure (IOP) is within normal limits (WNL). Extraocular muscles (EOM) were full, and the nystagmus has dampened over time. Anterior segments were WNL on repeated exams, with the appearance of mild early posterior subcapsular cataracts (PSC) opacity of the lens on her last exam. Fundus examination revealed characteristic signs of RP including optic disc pallor, mild attenuation of the retinal vessels, bone spicule-like pigmentary changes (bone spicule pigmentations [BSPs]), and grayish atrophy in the mid-peripheral retina. In the maculas, there were retinal pigment epithelium (RPE) changes associated with atrophy encroaching upon the foveas, mainly on the nasal and inferonasal aspects (Figure 3).

**FIGURE 4:**
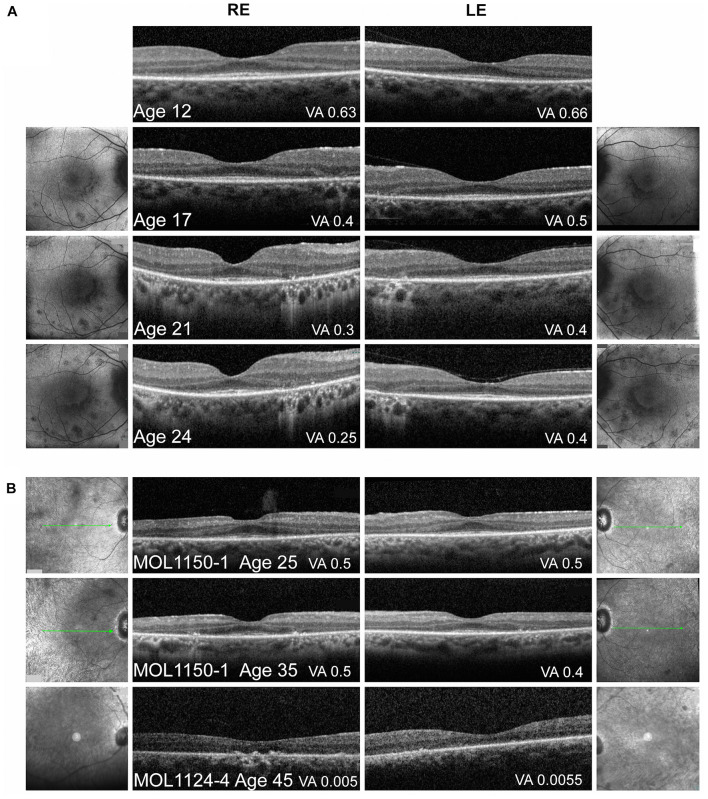
Macular imaging. **(A)** Representative macular FAF and foveal OCT images of MOL0358-1 over time. Thinning of the photoreceptor layer and narrowing of the central area of ONL and intact EZ can be observed with time. FAF imaging shows increase in the number and size of hypoautofluorescent spots in the macular area along time, some encircling the central macula, with a faint hyperautofluorescent ring around the fovea. The BCVA measured on the day of OCT imaging is specified in the right bottom corner of each scan. **(B)** Macular OCT images from patient MOL1150-1 at the ages of 25 and 35 again show increased thinning of the photoreceptor layer and loss of intact EZ width with age. In patient MOL1124-4 at the age of 45, ONL and EZ are practically lost. The BCVA measured on the day of OCT imaging is specified in the right bottom corner of each scan.

**FIGURE 5:**
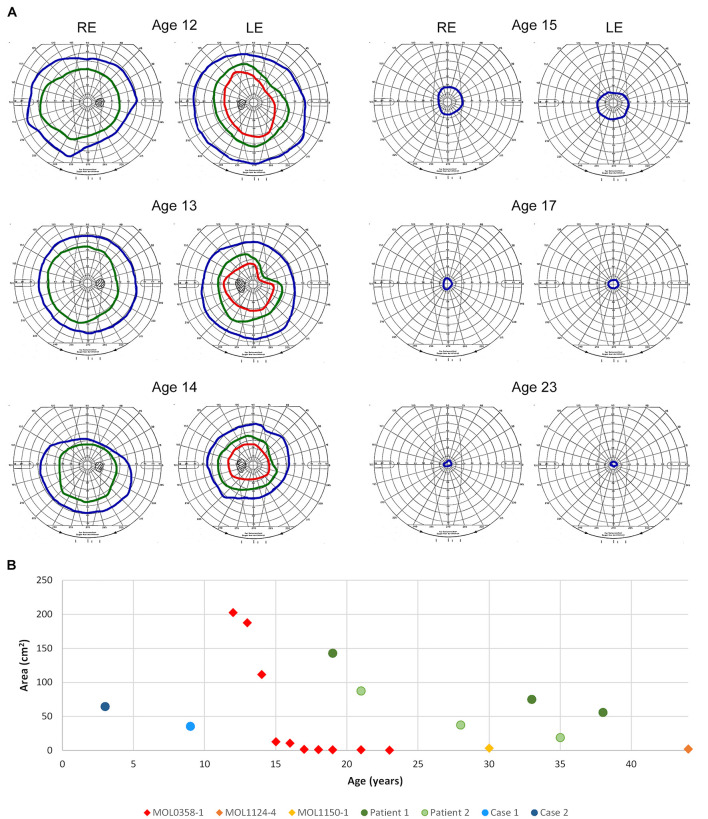
Goldmann kinetic perimetry. **(A)** Examples of VF from different ages of patient MOL0358-1. Target I4e shown in red, target III4e in green, and target V4e in blue. Rapid constriction of the VF is evident during teen years. **(B)** Visual field areas of MOL0358-1, MOL1124-4, MOL1150-1, and other patients from the literature (using target V4e) plotted versus age. Average VF of MOL0358-1 are presented in red rhombus. Average VF areas of other patients previously published in the literature are represented by circles: Green circles represent the VF of patients from the article by Sato et al. (2020), and blue circles represent the VF from Suzuki et al. (2014).

Optical coherence tomography imaging was performed almost every year since the age of 12, showing relative conservation of the photoreceptor layer and the ellipsoid zone (EZ) in the foveal area, with thinning and loss of the outer nuclear layer (ONL) and EZ outside the central macula. No cystoid macular edema (CME) was observed (Figure 4). Widefield FAF imaging shows hyperautofluorescence (hyperAF) around the arcades, with hypoautofluorescent (hypoAF) spots throughout the retina (Figure 3). Goldmann perimetry performed at the age of 12 showed relatively preserved VF. Subsequently, constriction of the VF was noted, with a significant drop in her mid-teens. At the age of 15, her VF were constricted to less than 20° from fixation using the V4e target in BE, and 2 years later, it dropped to 5° from fixation (Figure 5).

The mother of the index case (MOL0358-3) had a BCVA of 0.1 in the RE and 0.05 in the LE at the age of 39 (Figure 2), with horizontal nystagmus. Astigmatism was found in BE (Supplementary Table 2). IOP was 17 mmHg in BE, and a posterior polar cataract was noted. Funduscopy showed symmetric classic signs of RP with central, as well as peripheral, involvement including atrophy in the macular area, reddish-brown pigmentation in the foveal area, and BSP-like pigmentary changes in the periphery. ffERG responses were severely reduced in the RE and undetectable in the LE. Perimetry revealed very constricted VF, to less than 10° from fixation in BE.

One of the brothers of the index case (MOL0358-5) had a BCVA of 0.13 in BE at the age of 8 years (Figure 2). Fundoscopy showed symmetric disease with optic disc pallor, attenuated retinal vessels, and typical BSP-like changes in the equatorial and peripheral retinal regions. ffERG responses were severely reduced in BE, and VF were constricted to tunnel vision.

### MOL0597

Family MOL0597 is of Arab-Muslim origin, with multiple consanguineous marriages (Figure 1). The index case (MOL0597-1) and his sister (MOL0597-2) first presented to us at the age of 58 and 50 years, respectively, at a relatively advanced stage of disease. Both were homozygous for the c.2249A>G (p.Y750C) *RPGRIP1* mutation previously reported by us (Sharon et al., 2020; Figure 1).

At presentation, the index case had a BCVA of 0.33 Snellen in the RE and 0.50 in the LE (Figure 2), with a moderate hypermetropic and astigmatic correction (Supplementary Table 2). IOP was 16 mmHg in BE. The patient had mild PSC. Fundus examination revealed mild macular RPE changes and BSPs, as well as pigment clumps outside the arcades. ERG was undetectable in BE under all stimulus conditions.

The sister of the index case (MOL0597-2) had a BCVA of 0.10 Snellen in BE (Figure 2), with rotatory nystagmus. IOP was 12 mmHg in BE, and PSC were observed. Fundus examination revealed attenuated retinal vessels, macular atrophy, and diffusely scattered BSP-like pigmentary changes. ERG was undetectable.

### MOL0600

The index case (MOL0600-1) descends from an Arab-Muslim family and was clinically diagnosed with LCA early in life. First signs were noticed at the age of 1–2 months, and already at the age of 7 years, he had no LP in BE. ERG responses were undetectable at that time. This patient was identified to harbor a homozygous novel mutation in *RPGRIP1*, c.1145T>A (p.L382^∗^) (Figure 1).

### MOL1124

MOL1124 is a consanguineous family of Arab-Muslim origin (2:2, Figure 1). The index case (MOL1124-1) and his brother (MOL1124-2) were diagnosed with a cone-dominated disease, while a third brother (MOL1124-4) had more severe manifestations and was clinically diagnosed as LCA with prominent cone involvement. All affected brothers were homozygous for the c.2249A>G (p.Y750C) (Sharon et al., 2020) mutation, in similarity to family MOL0597 (Figure 1).

MOL1124-1 was noted to have photophobia and impaired VA from an early age, but presented to our electrophysiology service only at the age of 21 years. At that time, the patient had a BCVA of FC in BE (Figure 2). Cone responses were impaired, but isolated rod responses were WNL at that time. Interestingly, flash visual evoked potentials (Flash VEP) amplitudes were markedly reduced from the RE, and responses were non-detectable when the LE was stimulated. Of note, fundus exam was reported as normal. Retinal imaging was not performed.

The brother of the index case (MOL1124-2) was also noted to have photophobia and impaired VA from an early age, but presented to us only at the age of 33 years. At that time, BCVA in BE was 0.1 (Figure 2), with a high astigmatic correction (Supplementary Table 2). The anterior segments were WNL, and no cataract was noted. On fundus exam, optic discs, retinal vessels, and retinal periphery were WNL, but macular atrophy was noted. On ffERG testing, cone responses were undetectable, while isolated rod responses following dark adaptation were WNL. Flash VEP amplitudes were reduced also in this brother, with a normal implicit time.

The third brother in this family (MOL1124-4) was noted to have jerky nystagmus, visual impairment, and photophobia from birth. At the age of 5 years, he had a BCVA of 0.1 with an astigmatic correction of 3 diopters. BCVA deteriorated over time, and at the age of 44 years, his VA was at the level of HM (Figure 2 and Supplementary Table 2). PSC and nuclear sclerotic cataract (NS) also developed over time. Fundus exam at young ages was reported as normal, revealed pigment clumps in the periphery in his 20’s, and at the age of 45 and 48, the patient was noted to have attenuation of retinal vessels, widespread pigment clumps, and BSP-like pigmentary changes (Figure 3). Widefield FAF images at the age of 48 showed multiple hypoAF spots from the arcades to the periphery, with hyperAF in the macular areas. Small hypoAF spots were present in and around the foveas (Figure 3). OCT imaging revealed marked impairment of the outer retina, with loss of ONL and EZ (Figure 4). At the age of 28, ffERG responses were undetectable. Flash VEP responses were WNL in the RE and had reduced amplitudes with a normal implicit time in the LE. Goldmann perimetry at the age of 44 showed markedly constricted fields using the V4e target (Figure 5B).

### MOL1150

The index case (MOL1150-1) is an isolated case from a consanguineous family of Arab-Muslim origin (2:2; Figure 1). He was diagnosed with early-onset RP at the age of 5 years. Clinical details from that time are not available, but suspicion of disease arose because of poor night vision while VA was quite preserved. BCVA at the age of 25 was 0.50 in each eye, and this was maintained at least to the age of 35, at his last exam (Figure 2). His refraction was −3.25/−1.50 × 30° in the RE and −3.25/−1.50 × 164° in the LE. He also has a mild central PSC. At the age of 34 years, funduscopy revealed optic disc pallor and severe attenuation of retinal blood vessels, which were not observed outside the main arcade. The maculas were relatively preserved, and in the midperiphery, grayish atrophy with BSP-like pigmentary changes and pigment clumps were present. Widefield FAF imaging showed hyperfluorescence within the arcades with a hyperfluorescent ring around the foveas (Figure 3). On OCT at the age of 25, residual ONL and an intact EZ were seen in the foveal area in BE with thinning and loss of ONL in parafoveal areas. Ten years later, thinning and some constriction of the ONL islands occurred, and increased irregularity was noted in EZ (Figure 4). At the age of 19, photopic and scotopic FFERG responses were undetectable. Flash VEP responses were WNL in the RE and had mildly reduced amplitudes with a normal implicit time in the LE. This patient was identified with a homozygous mutation in *RPGRIP1*, c.2974delA (p.R992Efs^∗^9) (Figure 1).

### MOL1403

This family is an Ashkenazi Jewish family, with mixed origin (Russia, Poland, Hungary, and Germany) and no known consanguinity. The index case (MOL1403-1) and his brother (MOL1403-3) were diagnosed with CRD.

The index case had nystagmus and photophobia from early childhood. His BCVA at the age of 8 years was 0.10 in BE and remained the same for at least 10 years. His refraction was reported as +2.75 in BE at the age of 4 and +3.25/−100 × 8° in the RE and +4.50/−100 × 175° in the LE at the age of 15. No abnormal fundus findings were reported. On ffERG testing performed at the age of 4 years, rod responses were severely reduced, and cone responses were non-detectable. Flash VEP response was WNL in BE.

The brother of the index case (MOL1403-3) also had nystagmus from early childhood accompanied by mild photophobia. His BCVA at the age of 5 years was 0.30 in BE, and this was maintained also at his recent exam at age 11. His refraction was +1.25/−50 × 180° in the RE and +1.50/−25 × 180° in the LE at the age of 7. Fundus exam was reported as WNL. On ffERG testing at the age of 4.5 years, rod responses were moderately reduced, while his cones were non-detectable. The affected siblings from this family were identified to harbor the previously reported homozygous mutation, c.1615_1624del (p.E539Qfs^∗^2) (Sharon et al., 2020).

### TB33

In this consanguineous family (2:2) of Arab-Muslim origin, three siblings are affected (Figure 1). The index case (R73) had nystagmus and nyctalopia from childhood and was diagnosed with RP at the age of 9. At the age of 14, his BCVA was 0.50 (Figure 2). ffERG results showed severely reduced cone responses (RE 20 μV, 38 ms; LE 30 μV, 38 ms) and non-detectable rod responses.

His brother (R74) manifested nystagmus, nyctalopia, and myopia and was diagnosed with RP at the age of 8 years, with somewhat more severe disease than his older brother including constriction of the VF. At the age of 12, his BCVA was 0.4 (Figure 2), and his ERG was not detectable under both scotopic and photopic conditions.

A sister of those siblings (R77) first visited the clinic at the age of few months due to vertical nystagmus. She has not yet undergone ERG testing, but she and her two brothers were identified to manifest a homozygous RPGRIP1 mutation, c.2935C>T (p.Q979^∗^), which was previously reported by us (Sharon et al., 2020) as part of the Israeli consortium report (Figure 1).

In order to assess the spectrum of disease phenotypes associated with *RPGRIP1* mutations, we collected and summarized the clinical data provided for the 212 *RPGRIP1* patients reported in the literature and also combined the findings in our cohort of 16 patients. Out of 228 patients, the vast majority (197) were diagnosed with LCA, 18 with RP, and 13 with CRD. Approximately 80% of the patients (179 out of 228) are homozygous for the causative mutation, usually associated with consanguineous or intracommunity marriages.

### Signs and Symptoms

**FIGURE 6:**
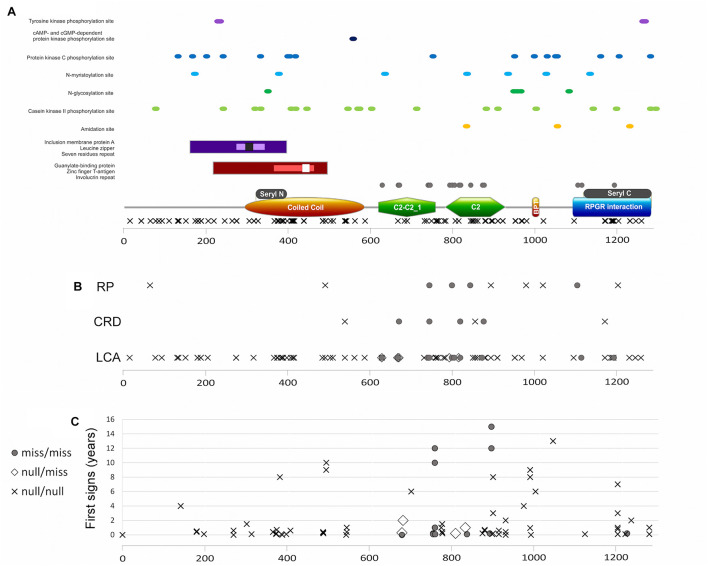
**(A)** RPGRIP1 protein structure including protein domains and functional sites and mutation distribution. Major protein domains (**A** bottom): orange rectangle represents the coiled coil domain, green polygon represents the C2-C2_1 and C2 domains, brown ellipse represents the bipartite nuclear localization domain, and blue rectangle represents the RPGR-interacting domain. Gray rectangles above the major domains represent the seryl tRNA synthetase N terminal and C terminal domains, respectively. Additional domains and areas: guanylate-binding protein marked with maroon rectangle, zinc-finger T-antigen with pink rectangle, involucrin repeat with white square, inclusion membrane protein A with purple rectangle, leucine zipper with lavender rectangle, and seven residues repeat with black square. Important sites marked with dots in different colors, as mentioned in the figure. Location of missense mutations is marked with gray circles above the major domains, and null mutations are marked with X below the major domains. **(B)** A plot of location of different mutation types along the protein and the clinical diagnosis of those patients. Gray circles represent the biallelic missense mutations, X represents the biallelic null mutations, and white rectangle represents the compound heterozygous condition with one missense and one null mutation. Frameshift or non-sense mutations that will lead to creation of prematurely truncated protein and are expected to abolish RPGRIP1 function are considered as null mutations. **(C)** A plot of appearance of first signs (as reported by the patient, in years) and location of the different mutation types along the protein.

Data regarding disease onset were available for 133 patients. First signs usually appeared in early childhood (as could be expected in LCA), with the vast majority of patients showing first signs before the age of 10 years (Figure 6C). The average age of first signs or symptoms was 1.7 ± 1.07 years.

Based on positive reports in the different publications, the prevalence of nystagmus among *RPGRIP1* patients is at least 45% (103 out of the 228 patients). At least 16% are photophobic (37 cases), and 12% showed an oculodigital sign (28 cases). In 12 patients (one diagnosed with RP and the rest with LCA), developmental delay, or intellectual disability was reported, but it is not clear whether of primary nature or secondary to the visual impairment. Of note, 3 out of 4 patients homozygous for the c.3565_3571delCGAAGGC mutation were reported to have intellectual disability (Miyamichi et al., 2019) as were the 2 patients with the c.1111C>T mutation (Zernant et al., 2005; Jinda et al., 2017).

### Refraction

Refractive data were available from 53 patients, and all but 6 were reported to be hyperopic. The 47 hyperopic patients (ages 1–39) manifested rather high hyperopia, with a mean spherical equivalent correction of +4.79+/−0.8D (SEM). In the four patients reported with high myopia (Tiwari et al., 2016; Imani et al., 2018), the exact refractive error was not specified, but interestingly, they all shared the same unique homozygous mutation, c.2889delT (p.Ser964fs), that did not appear in any other *RPGRIP1* patients. These four myopic patients belong to two different families originating from Turkish and Iranian descent.

### Visual Acuity

We collected data from 124 patients who had VA results, 112 from the literature and 12 from our cohort (Figure 2). When multiple measurements were available in a given patient, the earliest one was taken for the purpose of analysis of mean BCVA. Average Snellen VA was 0.06 ± 0.04 (SEM), with the mean age of measurement being 12.8 ± 4.4 years (SEM). Most of the patients performed their first measurement of VA at an early age, usually before the age of 10 years, and in most cases, rather low acuity was present (FC, HM, LP, or NLP). Only 17 patients had a VA better than 0.1 at the time of initial measurement, and, as can be expected, many of them were not clinically diagnosed with LCA.

### Fundus Findings

We summarized all the reported observations from previous published papers that included any fundus description (Dryja et al., 2001; Chen et al., 2013; Fakhratova, 2013; Khan et al., 2013, 2014; Suzuki et al., 2014; Saqib et al., 2015; Wang et al., 2016; Han et al., 2017; Huang et al., 2017; Trichonas et al., 2017; Birtel et al., 2018; Hosono et al., 2018; Imani et al., 2018; Miyamichi et al., 2019; Sato et al., 2020). Variable funduscopic findings were reported for LCA patients harboring mutations in *RPGRIP1*, but in most cases, at early ages, findings were relatively mild (Khan et al., 2013; Suzuki et al., 2014). Over time, usually during the first and second decades of life, grayish spots and gray areas of atrophy appeared, along with RPE mottling and gradual attenuation of vessels. Later, migration of pigment that in some cases forms bone spicule-like changes or pigment clumps appears along with waxy pallor of the optic discs (Dryja et al., 2001; Fakhratova, 2013; Khan et al., 2013; Huang et al., 2017; Birtel et al., 2018; Miyamichi et al., 2019). Some patients demonstrate macular involvement concomitant with the peripheral retinal atrophy (Maria et al., 2015; Saqib et al., 2015). In FAF images, hyperAF is noticeable in the macular area, surrounded by hypoAF areas. Peripheral hypoAF areas appear and extend at later stages of the disease. Examples of fundus photos and FAF images of patients from our cohort are presented in Figure 3.

### Macular Optical Coherence Tomography, Fundus Autofluorescence, and Infrared Imaging

An overview of OCT images from the literature showed thinning of the retina accompanied by disruption of the ONL, EZ, and RPE layers as the disease progressed (Han et al., 2017; Imani et al., 2018; Miyamichi et al., 2019). Representative OCT images of *RPGRIP1* patients from the Israeli cohort at different ages are presented in Figure 4. Repeated OCT imaging performed over 12 years in patient MOL0358-1 documents structural changes in the macular area during the period in which a major drop in VA and constriction of the VF occurred, correlating with gradual diminution of the central area of retained ONL and intact EZ (Figure 4A). Macular FAF imaging at the same time points (on both sides of the OCT scans) shows gradual increase in the number and size of hypoAF spots around the foveas. OCT imaging in patient MOL1150-1 performed a decade apart, at the ages of 25 and 35, shows similar disease progression (Figure 4B, two upper scans). At the age of 45, OCT scans in patient MOL1124-4 reveal practically total loss of ONL and EZ, as well as disruption of the outer retina/RPE layers with hyperreflective foci in the RE (Figure 4B, lowest panel). Interestingly, none of our patients showed cystoid changes or macular edema, and CME was also not reported among *RPGRIP1* patients in the literature.

### Visual Fields

Perimetry results in *RPGRIP1* patients were infrequently reported in the literature, probably due to the early age of onset and severe impairment of the VF from early ages that precludes accurate measurements. Among the 212 patients reported in the literature, we were able to collect only 12 reports of Goldmann VF that were measured using the V4e target, but most were reported without an accompanying figure (Suzuki et al., 2014; Sato et al., 2020). In a few additional patients, VF were measured using the Octopus perimeter, and in some manuscripts, constricted VF were reported without specifying the method used or the actual results of the measurements. In our cohort, serial Goldmann perimetry in patient MOL0358-1 shows rapid constriction of the VF during her teen years, to less than 7° from fixation using the V4e target at age 17 (Figure 5A). The course of deterioration in the VF area with age in this patient is shown by red rhombi in Figure 5B, along with two more patients from our cohort who at the ages of 30 and 45 years had only very small central islands remaining (yellow and orange rhombi). Goldmann VF areas in four additional patients reported in the literature (from two different manuscripts) are also plotted and represented by circles.

### Full-Field Electroretinography

Electroretinography testing results were available for 25/212 patients reported in the literature and in 14/16 patients in our cohort. In the vast majority of cases, testing was performed at very early ages, as part of the workup of babies and young children manifesting severe visual impairment (mean age at ERG testing 8.5 ± 2.5 years). In most cases (27/39), both cone and rod ffERG responses were non-detectable already at the time of first testing, and only in one case, rods were undetectable, while cone responses were severely reduced. In the remaining 10 cases, cone responses were non-detectable, but some residual rod responses, while often very severely reduced, were still measurable. Of note, two siblings from family MOL1124 of our cohort (MOL1124-1 and MOL1124-2) diagnosed with CRD had at age 21 and 33, respectively, non-detectable cone responses, but isolated rod responses were WNL(!) (Supplementary Table 2). Interestingly, these brothers carried the c.2249A>G (p.Y750C) mutation in the homozygous state. In two siblings with the same homozygous mutation from another family (MOL0597-1 and MOL0597-2), the course of disease is presumed to also be exceptionally mild, as they were diagnosed only when they reached their 40’s or 50’s. Except these rare patients, according to the ERG results, the majority of *RPGRIP1* patients indeed manifest a LCA phenotype with extinguished ERG responses at the time of first testing, while a minority, when examined at very early ages, manifest severe retinal degeneration with a cone–rod pattern of disease expression.

### Genetics and Mutations

A total of 122 biallelic disease-causing mutations in *RPGRIP1* have been reported thus far in the literature. The most frequently reported mutations were c.1107delA (p.Glu370Asnfs^∗^5) that was reported in 25 LCA patients, c.2480G>T (p.Arg827Leu) in 12 patients (most of whom were diagnosed with LCA and some with CRD), and c.2236G>A (p.Gly746Arg) in 7 LCA patients.

**FIGURE 7:**
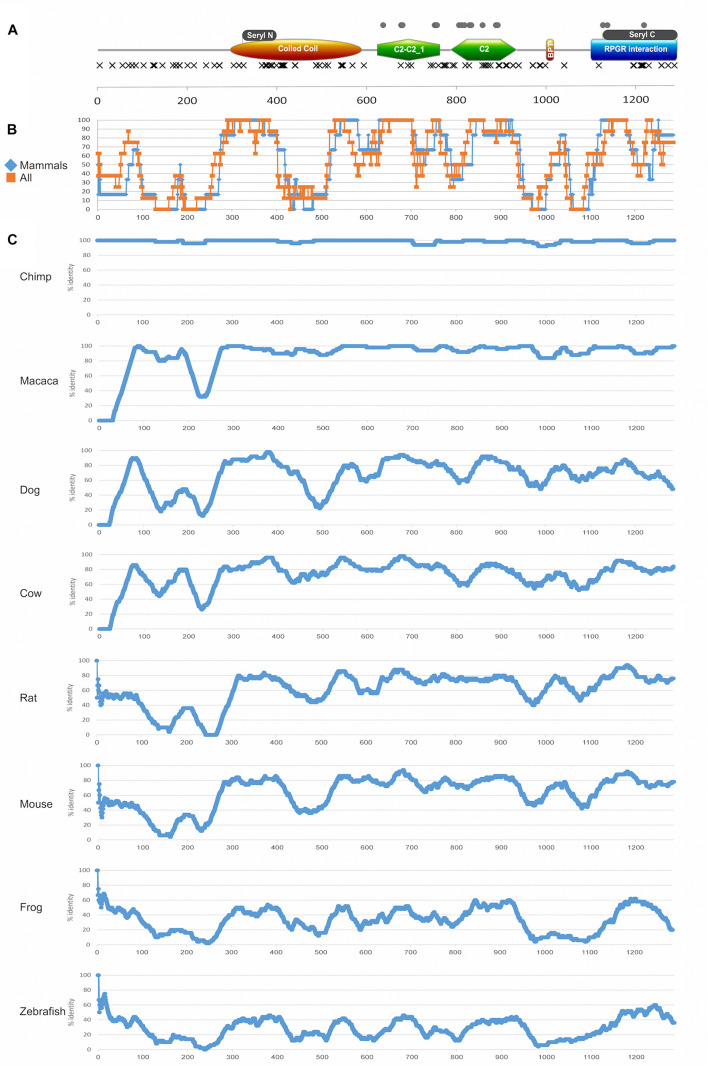
Protein domains and evolutionary analysis of *RPGRIP1*. **(A)** A schematic representation of the RPGRIP1 protein. Orange ellipse represents the coiled coil domain, green polygon represents the C2-C2_1 and C2 domains, orange rectangle represents the bipartite nuclear localization domain, and blue rectangle represents the RPGR-interacting domain. Gray rectangles above the major domains represent the seryl tRNA synthetase N-terminal and C-terminal domains, respectively. Frameshift and nonsense mutations are expected to result in a prematurely truncated protein and are therefore considered as null mutations and marked with black X that are located underneath major domains. Missense mutations are marked with gray circles and are located above the major domains. **(B)** A summary of amino acid sliding window (length of 50 amino acids) comparing the human protein sequence to selected orthologs (chimp, macaca, dog, cow, rat, mouse, frog, and zebrafish) is presented. Average of only mammalian sequences (blue) versus all the sequences (orange) is presented. **(C)** An amino acid sliding window comparing the human protein sequence to each ortholog (chimp, macaca, dog, cow, rat, mouse, frog, and zebrafish) is shown. *X*-axis: amino acid number; *Y*-axis: percentage of amino acid identity in a 50 amino acid window. Accession numbers are as follows: human (NP_065099.3), chimpanzee (PNI22424.1), macaca (XP_028707222.1), dog (NP_001300702.1), cow (XP_024853050.1), rat (XP_017455358.1), mouse (NP_076368.1), frog (XP_012826764.2), and zebrafish (XP_021325946.1). The average percentage of amino acid identity for each studied sequence: chimp 98%, macaca 95%, dog 70%, cow 77%, rat 61%, mouse 64%, frog 33%, and zebrafish 29%.

Most of the mutations reported so far are nonsense or splicing mutations that are predicted to be null (101 different mutations representing 363 alleles). The remaining 21 mutations are missense (93 alleles). We collected from the literature data regarding all previously reported mutations and presented them according to their location on the protein sequence (Figure 7A). The analysis shows that null mutations are spread all along the protein, while missense mutations occur only in three protein domains: C2-C2_1 domain, C2 domain, and RPGR-interacting domain (Figure 7A). According to the scattering of the mutations along the protein, no missense mutations were detected before C2-C2_1 domain, indicating that missense changes in this area are likely to be tolerated.

A sliding window analysis of RPGRIP1 orthologs was performed aiming to gain insight into the conservation pattern of RPGRIP1 along evolution (Figures 7B,C). The analysis highlights a few highly preserved regions that correspond to protein domains, including the seryl tRNA synthetase N-terminal domain, the end of the coiled coil region, the bipartite nuclear localization domain, the C2-C2_1 and the C2 domains, and the seryl tRNA synthetase C-terminal domain. On the other hand, two regions that are part of known domains showed a relatively low level of preservation: the coiled coil domain with an exception of seryl tRNA synthetase N-terminal domain (amino acids (aa) 395–524, Figure 7B), where no missense mutations were identified, and the RPGR-interacting domain (aa 1086–1286), which is less conserved in the upstream region, and no missense mutations were identified in this region.

In addition, one can note that in the coiled coil domain (aa 294–584) that includes the seryl tRNA synthetase N-terminal domain (aa 321–384), only null mutation was reported. The C2-C2_1 domain, which is located between aa 619 and 757, contains 19 mutations (a total of 111 reported mutated alleles), 9 of which are null and 10 are missense (Figure 7A). This area is highly conserved among all species (Figures 7B,C). The second C2 domain (aa 781–927) contains 23 mutations (a total of 40 alleles), most of which (15 mutations) are null. However, while the null mutations are spread along the whole C2 domain, all missense mutations are concentrated in the middle region of this domain (aa 781–886), and the region is highly conserved (Figure 7B). The RPGR-interacting domain (aa 1086–1286) is the most C-terminal domain and it includes the seryl tRNA C-terminal domain (aa 1117–1282). The seryl tRNA C-terminal domain seems to be much less sensitive to missense changes. A total of 11 mutations (reported in a total of 65 alleles) were reported in this domain, most of which (8) are null.

### A Brief Overview of the Reported Mutations and Phenotypes

To date (including the cases reported here), 228 biallelic *RPGRIP1* cases have been reported. Of which, 41 patients had biallelic missense mutations (21 were diagnosed with LCA, 15 with CRD/CD, and 5 with RP – all non-syndromic), 5 were compound heterozygous for a null and a missense mutation (all diagnosed with non-syndromic LCA), and the remaining 182 cases had two null mutations (163 of whom were diagnosed with LCA, 13 with RP, and 6 with CRD). Interestingly, intellectual disability (with or without reported developmental delay) has been reported in 12 out of the 228 cases, all of whom had homozygous biallelic null mutations.

Since three different IRD phenotypes have been reported due to *RPGRIP1* mutations, accompanied by intellectual disability in the minority of cases, we carried out a comprehensive genotype–phenotype analysis, including information on all 228 reported cases aiming to identify potential correlations between mutation type and/or location and clinical features such as phenotype and age of onset. We therefore performed a protein structure analysis (Figure 6A) to better characterize relatively small active sites (such as tyrosine kinase phosphorylation sites, cAMP- and cGMP-dependent protein kinase phosphorylation sites, protein kinase C phosphorylation sites, N-myristoylation sites, N-glycosylation sites, casein kinase II phosphorylation sites, amidation sites), as well as larger repeats and domains (including leucine zipper, inclusion membrane protein A, seven residue repeat, guanylate-binding protein, zinc finger large T-antigen D1-type profile, and involucrin repeats areas). The analysis did not reveal any missense mutations within or in close proximity to the above-mentioned sites (Figure 6A).

Subsequently, we performed a Chi-square analysis that is based on the distribution of the three different phenotypes between the three possible genotypes (biallelic null, biallelic missense, and compound heterozygous for a null and a missense mutation). The analysis revealed a significant difference in the distribution of CRD cases among the different genotypes (*p* = 1.47^∗^10^–9^) with a lower than expected number of CRD cases in the null/null group and a higher number of cases in the missense/missense group. Similarly, but with a borderline significance value (*p* = 0.04), LCA cases were more common in the null/null group and less common in the missense/missense group (Supplementary Figure 1). No significant difference was obtained for RP.

Interestingly, some of the reported mutations show variability in the resulting phenotype, even when they appear as homozygous (e.g., homozygosity for c.1468-2A>G and c.2417C>T can lead to either LCA or RP, c.2480G>T and c.3565C>T to LCA or CRD, and c.2249A>G to either LCA, RP, or CRD).

Data regarding disease onset were available for 133 patients. First signs appeared usually in early childhood (the vast majority of patients experienced first signs earlier than the age of 10 years, Figure 6C), with an average age of first signs or symptoms of 1.7 ± 1.07 years. Correlation analysis between genotype (missense/missense, null/missense, and null/null) and age of onset (Figure 6C) did not reveal a significant difference.

## Discussion

In this article, we summarize and present clinical data of 228 patients with biallelic *RPGRIP1* mutations. Sixteen of those patients belong to the Israeli cohort and are presented in this study for the first time. The remaining patients, to the best of our knowledge, reflect all *RPGRIP1* cases described in the literature up to the cutoff date of our search that was January 31, 2021. Out of 228 patients, 179 are homozygous for the disease-causing mutation, indicating probable consanguinity or intracommunity marriages in the background. Patients with biallelic *RPGRIP1* mutations are usually reported to express a LCA phenotype (197, 86%), but also RP (18, 7%) and CRD (13, 5%) phenotypes were described. In the Israeli cohort of patients, the phenotype tends to be milder, with most of the patients clinically diagnosed as RP (10, 62.5%) or CRD (4, 25%) and only a minority reported to have LCA (2, 12.5%). These three phenotypes essentially lie along a spectrum and may overlap in their clinical appearance, especially if seen at different stages of disease, such that the distinction between them is not always clear. Our impression is that LCA with prominent cone involvement is the phenotype that best describes the majority of cases.

Worldwide, 5–7% of LCA cases are reported to be caused by mutations in *RPGRIP1* (Dryja et al., 2001; Gerber et al., 2001; den Hollander et al., 2008). In the Israeli cohort of patients, out of 144 patients (112 families) with a LCA phenotype (of which 94 families are genetically diagnosed), approximately 1.5% of cases (2 patients) are due to biallelic *RPGRIP1* mutations. However, there is a higher prevalence of *RPGRIP1* patients who manifest milder disease in our cohort (14/16 patients). As will be detailed below, this does not seem to be related to the prevalence of missense versus null mutations among our patients.

Interestingly, different clinical phenotypes (LCA, RP, or CRD) can appear in families with the same homozygous mutation and even within the same family, as is also reported here. For example, MOL0597 and MOL1124 (both of Arab-Muslim descent) have the same homozygous c.2249A>G (p.Y750C) mutation with two siblings in MOL0597 manifesting RP, while in MOL1124, two brothers manifest CRD, and the third manifests LCA. In the literature, we were able to identify additional mutations that are associated with variable phenotypes, either within the same article or reported in different articles. These include c.1306G>T (p.A436S) associated with LCA or CRD (Tayebi et al., 2019), c.2417C>T (p.T806I) reported with LCA or RP (Vallespin et al., 2007), c.2480G>T (p.R827L) reported with LCA (McKibbin et al., 2010) or CRD (Hameed et al., 2003) in two different articles, and c.1468-2A>G as the cause of LCA (Li et al., 2011) or RP (Huang et al., 2017). Such differences in clinical diagnosis may be explained by human factor – examination by different clinicians in different centers, located in different countries, and perhaps at different ages of the patients. However, there are cases when patients were examined in the same center by the same clinicians and still received different clinical diagnoses. Inter- and intra-familial variability of disease expression even when the same gene and mutation are present is not rare in IRDs in general, and common explanations for this phenomenon include possible modifying genetic and environmental factors. For example, in the case of *RPGR* (which also interacts with *RPGRIP1*), mutant mice from different genetic backgrounds that share the same mutation demonstrated a very different course of disease: a rod-dominated phenotype was observed in mice on a BL/6 background and cone-dominated disease was observed for the same mutation in BALB/c mice (Brunner et al., 2010). Specifically in the case of *RPGRIP1*, a number of studies probed this issue of phenotypic variability in the canine model of Rpgrip1 retinal degeneration, in which different ages of onset and variable severity of disease are seen in dogs with the same *RPGRIP1* mutation (Miyadera et al., 2009; Forman et al., 2016; Das et al., 2017). Forman and colleagues hypothesized that differences in age of onset may be explained by modifier genes such as *MAP9* (Forman et al., 2016). Das et al. (2017) suggested a multigenic explanation for the phenotypic variability. Miyadera et al. (2009) demonstrated leakiness of the frameshift mutation present in these dogs that may also explain the variable appearance of disease in Rpgrip1^–/–^ dogs. Interestingly, some of the mutations listed above associated with different phenotypes in human patients are also frameshift mutations that may lead to splicing errors, and perhaps similar “leakiness” can explain the difference in disease severity among these patients.

Evolutionary analysis of the RPGRIP1 protein in different species led to the identification of highly conserved regions in the three domains in which missense changes were pathogenic. We identified additional highly conserved regions in protein domains (including the N-terminal seryl t-RNA synthetase domain, the end of the coiled coil domain, and the bipartite nuclear localization domain), but no missense mutations were reported in these regions. This can be explained by retained partial protein activity in alleles that bear missense mutations or even in-frame deletions, as reported to be the case for the N-terminal seryl tRNA synthetase domain that was studied in *Escherichia coli* (Vincent et al., 1995). None of the missense mutations was identified in biochemically active sites (19 casein kinase II phosphorylation sites, 17 protein kinase C phosphorylation sites, 7 N-myristoylation sites, 5 N-glycosylation sites, 3 amidation sites, and 2 tyrosine kinase phosphorylation sites), which might indicate overlapping functions of these sites.

Previous publications (Li, 2015; Huang et al., 2017; Miyamichi et al., 2019) suggested that the LCA phenotype is usually caused by premature termination codons or null alleles, while a less severe form of the disease, CRD, is associated with missense mutations that retain partial function of the protein. After preforming Chi-square analysis on possible phenotypes and genotypes, we indeed identified significant differences in the distribution of CRD and LCA cases. CRD phenotypes were more common in the biallelic missense group and much less common in the biallelic null group, while LCA phenotypes showed the opposite pattern. This finding indicates that two null alleles tend to cause more severe disease than two missense alleles. It should be noted that the patients included in this article were diagnosed in many different centers and they were examined at different stages of their disease, factors that could influence the way their phenotype was defined.

Intellectual disability or developmental delay was reported in 12 patients with RPGRIP1 disease (Zernant et al., 2005; Khan et al., 2013, 2014; Abouzeid et al., 2016; Jinda et al., 2017; Sanchez-Navarro et al., 2018; Jespersgaard et al., 2019; Miyamichi et al., 2019). Interestingly, two specific RPGRIP1 mutations (c.3565_3571delCGAAGGC – three patients from two families and c.1111C>T – two patients from two families) were reported only in patients with intellectual disability or developmental delay. Recessive mutations in RPGRIP1L, which is a homolog of RPGRIP1, can cause systematic ciliopathies like Joubert syndrome-7 (Delous et al., 2007), Meckel syndrome 5 (Delous et al., 2007), and COACH syndrome 3 (Doherty et al., 2010), which also involve intellectual disability. RPGRIP1L and RPGRIP1 are known to interact with the NPHP4 C2 domain (Roepman et al., 2005; Arts et al., 2007), and mutations in both can cause developmental delay by interfering with neuronal development (Wang et al., 2019). It is possible that developmental delay or intellectual disability in patients with *RPGRIP1* mutations is caused by the same molecular pathway. Alternatively, since all 12 patients with intellectual disability or developmental delay had homozygous mutations, we cannot exclude the possibility that underlying consanguineous or intracommunity marriages elevate the risk for intellectual disability caused by other recessive inherited causes.

Most of the patients reported first signs of disease very early in life, compatible with the majority being diagnosed with LCA. While low vision was present in practically all patients, many signs and symptoms were not constitutive in their appearance. For example, nystagmus and oculodigital signs do not appear in all patients. While most of the patients are hyperopic (Dryja et al., 2001; Galvin et al., 2005; Fakhratova, 2013; Khan et al., 2013, 2014; Verma et al., 2013; Suzuki et al., 2014; Wang et al., 2016; Han et al., 2017; Huang et al., 2017; Sato et al., 2020), some myopic patients were identified (Tiwari et al., 2016; Imani et al., 2018); most of the patients reported light aversion (McKibbin et al., 2010; Fakhratova, 2013; Huang et al., 2013; Khan et al., 2013, 2014; Suzuki et al., 2014; Saqib et al., 2015; Wang et al., 2016; Sato et al., 2020; Skorczyk-Werner et al., 2020), but some emphasize nyctalopia as a major visual symptom (McKibbin et al., 2010; Birtel et al., 2018; Miyamichi et al., 2019). Hanein et al. (2004) suggested a clinical flowchart according to which a child manifesting nystagmus, profound visual deficiency, and unrecordable ERG at birth or during the first months of life and developing photophobia, showing hypermetropia with a refractive error lower than +7, and VA better than FC, but worse than 1/20, will probably have RPGRIP1 or AIPL1 mutations. While this flowchart holds true for many of the 228 RPGRIP1 patients included in the present analysis (from multiple centers and publications), not all patients with *RPGRIP1* causative mutations have nystagmus from birth (156 patients, which are 68%), a minority still have recordable ERG responses even after the age of 10 years, approximately 25% of patients are nyctalopic, 20% (25/124, 7 of them had NLP) of patients had VA lower than FC (Dryja et al., 2001; Galvin et al., 2005; McKibbin et al., 2010; Walia et al., 2010; Chen et al., 2013; Fakhratova, 2013; Huang et al., 2013, 2017; Khan et al., 2013, 2014; Wang et al., 2016; Han et al., 2017; Jinda et al., 2017; Hosono et al., 2018; Weisschuh et al., 2018; Avela et al., 2019; Jamshidi et al., 2019; Sallum et al., 2020; Skorczyk-Werner et al., 2020), and 25% (32/124) had a VA better than 1/20 (McKibbin et al., 2010; Fakhratova, 2013; Suzuki et al., 2014; Saqib et al., 2015; Han et al., 2017; Huang et al., 2017; Birtel et al., 2018; Weisschuh et al., 2018; Imani et al., 2018; Miyamichi et al., 2019; Skorczyk-Werner et al., 2020; Sallum et al., 2020; Sato et al., 2020). In addition, there are patients who are myopic (Tiwari et al., 2016; Imani et al., 2018) or have hypermetropia higher than +7 (Galvin et al., 2005; Khan et al., 2013, 2014). Approximately 55% of the patients indeed had VA within the range suggested by Hanein et al. (2004) usually measured early in life (before the age of 10). A wide spectrum of findings is not uncommon in many inherited retinal degenerations.

Despite low VA and appearance of other signs and symptoms early in life, fundus findings at infancy and early childhood are relatively mild (as previously reported in the literature and summarized by us), and appearance of pigmentary changes or grayish spots of atrophy occurs in late childhood or teen years, with BSP-like pigmentary changes appearing even later in life. According to all the collected reports from the literature, thinning of the retina accompanied by disruption of the ONL appears from early stages of the disease, later also involving the EZ. Complete degeneration of the ONL and total disruption of the EZ do not appear at a defined age range and differ from patient to patient according to the course of disease.

Few perimetry results of RPGRIP1 patients are reported in the literature. VF that are done in different centers using different methods are not easily comparable, but in general, reported VF in RPGRIP1 patients are constricted from early ages, with few exceptions such as case MOL0358-1 described above in which VF deteriorated during her teen years.

Electroretinography results were available for only 39 patients: most patients indeed manifest a LCA phenotype with extinguished ERG responses, while a minority, when examined at very early ages, manifest severe retinal degeneration with a cone–rod pattern of disease expression.

In summary, after reviewing all previously published papers, it appears that mutations in the *RPGRIP1* gene can cause severe retinal degeneration, which is usually characterized by rapid deterioration in visual function during the first years of life. However, very few patients (7/124 for which VA was reported) reach NLP. Thus, most of the patients preserve some (usually low) VA for a long period (several decades), indicating that some functional photoreceptors still exist and could potentially be a target for gene augmentation therapy. Recent publications on successful gene therapy in mouse and dog models of RPGRIP1 retinal degeneration were published (Pawlyk et al., 2010; Lhériteau et al., 2014) and demonstrated preservation of retinal function in treated areas. In the mouse model, survival of photoreceptors, preservation of their morphology, and better preservation of ERG responses were reported (Pawlyk et al., 2010). In dogs, improved structure and function was reported even 24 months after subretinal injection of adeno-associated viruses (AAV) vector carrying the *RPGRIP1* gene (Lhériteau et al., 2014). RPGRIP1 patients who still have evidence for retained ONL on OCT imaging may stand to benefit from similar therapy.

## Data Availability Statement

The original contributions presented in the study are publicly available. This data can be found here: BioProject PRJNA752818.

## Ethics Statement

The studies involving human participants were reviewed and approved by the Hadassah-Hebrew University Medical Center and the Rambam Health Care Center IRB Committees. Written informed consent to participate in this study was provided by the participants’ legal guardian/next of kin. Written informed consent was obtained from the individual(s), and minor(s)’ legal guardian/next of kin, for the publication of any potentially identifiable images or data included in this article.

## Author Contributions

AB conceived the topic, prepared the figures, and wrote the manuscript. AB, HA, RC, MB, OI, RA, SK, TB-Y, and EB collected and analyzed the clinical data. AB, FC, MK, AS, TB-Y, and DS performed the genetic analysis. DS and EB edited the manuscript and the figures. All authors read the final version of the article and approved it.

## Conflict of Interest

The authors declare that the research was conducted in the absence of any commercial or financial relationships that could be construed as a potential conflict of interest.

## Publisher’s Note

All claims expressed in this article are solely those of the authors and do not necessarily represent those of their affiliated organizations, or those of the publisher, the editors and the reviewers. Any product that may be evaluated in this article, or claim that may be made by its manufacturer, is not guaranteed or endorsed by the publisher.

## Footnotes

1
https://sph.uth.edu/retnet/

2
http://www.bioinformatics.nl/cgi-bin/primer3plus/primer3plus.cgi/

3
http://genetics.bwh.harvard.edu/pph2/

4
http://www.mutationtaster.org/

5
http://sift.jcvi.org/

6
https://www.myvisiontest.com

7
http://www.ncbi.nlm.nih.gov/homologene

8
http://www.ebi.ac.uk/Tools/clustalw2/index.html

## Abbreviations

AAV: adeno-associated viruses
BCVA: best corrected visual acuity
BE: both eyes
BSPs: bone spicule pigmentations
CME: cystoid macular edema
CRD: cone–rod dystrophy
EOM: extraocular muscles
EZ: ellipsoid zone
FAF: fundus autofluorescence
FC: finger count
ffERG: full-field electroretinography
Flash VEP: flash visual evoked potentials
HM: hand movement
hyperAF: hyperautofluorescence
hypoAF: hypoautofluorescent
IOP: intraocular pressure
IRDs: inherited retinal dystrophies
LCA: Leber congenital amaurosis
LE: left eye
LP: light perception
MIPs: molecular inverted probes
NLP: no light perception
NS: nuclear sclerotic cataract
OCT: optical coherence tomography
ONL: outer nuclear layer
PSC: posterior subcapsular cataracts
RE: right eye
RP: retinitis pigmentosa
RPE: retinal pigment epithelium
VA: visual acuity
VF: visual fields
WES: whole exome sequencing
WNL: within normal limits.
